# Cognitive and emotional profiles in children with ASD, ADHD, and comorbid presentations: evidence for a distinct clinical phenotype

**DOI:** 10.3389/fpsyt.2026.1765698

**Published:** 2026-03-03

**Authors:** Antonio Narzisi, Federica Barbetti, Maddalena Fabbri-Destro, Stefano Berloffa, Pamela Fantozzi, Valentina Viglione, Rosy Muccio, Elena Valente, Ilaria Accorinti, Elisa Foti, Annarita Milone, Ramona Cardillo, Gabriele Masi

**Affiliations:** 1 Department of Child Psychiatry and Psychopharmacology, IRCCS Foundation Stella Maris, Pisa, Italy; 2 Consiglio Nazionale Delle Ricerche, Istituto di Neuroscienze, Parma, Italy; 3 Department of Developmental Psychology and Socialization, University of Padua, Padua, Italy

**Keywords:** ADHD, ASD, behavioral dysregulation, CBCL, cognitive profile, comorbidity, executive functioning, WISC-IV

## Abstract

**Background:**

Autism Spectrum Disorder (ASD) and Attention-Deficit/Hyperactivity Disorder (ADHD) frequently co-occur, yet their comorbid presentation (ASD+ADHD) remains under- characterized. Clarifying cognitive and behavioral profiles is crucial for accurate diagnosis and effective intervention.

**Methods:**

A total of 207 children and adolescents (ages 6–16) were assessed using the Wechsler Intelligence Scale for Children – Fourth Edition (WISC-IV) and the Child Behavior Checklist (CBCL 6–18). Participants were grouped into ASD (n = 21), ADHD (n = 103), and ASD+ADHD (n = 83) cohorts. Group differences were analyzed through ANOVAs with Bonferroni corrections; Pearson correlations explored associations between cognitive indices and behavioral outcomes.

**Results:**

Children with ASD+ADHD scored significantly lower than the ASD group in working memory, processing speed, and full-scale IQ, while no significant differences emerged between the ASD+ADHD and ADHD groups. Behaviorally, ADHD participants exhibited higher externalizing symptoms (e.g., aggression, rule-breaking), while the ASD group showed greater withdrawn/depressed traits. The comorbid group presented the broadest dysregulation, with elevated scores across both internalizing and externalizing domains, including Sluggish Cognitive Tempo and obsessive-compulsive symptoms. Notably, protective associations between cognitive abilities and behavioral regulation, present in ASD and ADHD, were absent in the ASD+ADHD group.

**Conclusions:**

Findings suggest that ASD+ADHD comorbidity represents a distinct clinical profile, marked by compounded cognitive impairments and pervasive emotional-behavioral dysregulation. These patterns underscore the need for differential diagnostic approaches and tailored interventions that account for the unique neurocognitive architecture of comorbid presentations.

## 1 Introduction

Autism Spectrum Disorder (ASD) and Attention-Deficit/Hyperactivity Disorder (ADHD) are among the most prevalent neurodevelopmental disorders in childhood and adolescence, often associated with significant impairments in cognitive, emotional, and adaptive functioning (1–4). While traditionally considered separate diagnostic entities, recent literature has increasingly emphasized the substantial overlap between ASD and ADHD in both symptom expression and underlying neurobiological mechanisms (5–7). To better conceptualize how ASD and ADHD can co-occur, several theoretical models of comorbidity have been proposed. Antshel and Russo (7) describe three main frameworks: the splitter model, which views ASD and ADHD as distinct disorders that frequently co-occur due to partially shared etiological factors; the subgroup model, which posits that individuals with ASD+ADHD represent a specific subgroup within one or both conditions characterized by unique neurocognitive features; and the potentiation model, according to which the simultaneous presence of ASD and ADHD leads to interactive or synergistic effects, resulting in more severe or qualitatively different clinical presentations. These models highlight that comorbidity may reflect not only additive symptomatology but also interactive mechanisms shaping developmental trajectories. In parallel, increasing attention has been devoted to sensory processing characteristics in both ASD and ADHD. Sensory atypicalities are well documented in ASD and are now recognized as a diagnostic feature, often involving hyper- or hypo-reactivity to sensory input and atypical sensory seeking. However, growing evidence indicates that sensory processing differences are also highly prevalent in ADHD, particularly in the domains of tactile, auditory, and visual modulation. Recent work (8) suggests that while ASD is more often associated with pervasive sensory modulation differences, ADHD may present with context-dependent sensory regulation difficulties linked to attentional control and arousal systems. Importantly, children with co-occurring ASD and ADHD may exhibit more complex sensory profiles, potentially reflecting the interaction between atypical perceptual processing and regulatory control mechanisms. Considering sensory processing may therefore be essential for understanding the heterogeneity of ASD+ADHD presentations.

Epidemiological estimates suggest that up to 70% of children with ASD also meet criteria for ADHD, while 30–50% of those with ADHD show subclinical or clinical autistic traits (9, 10).

Despite their high co-occurrence, ASD+ADHD presentations remain poorly understood and frequently underdiagnosed. This has direct clinical consequences, including misclassification, inadequate treatment targeting, and increased burden on families and educational systems (7, 11). There is therefore a growing need to identify whether ASD+ADHD comorbidity represents a discrete neurodevelopmental phenotype or simply the additive effect of two overlapping syndromes (12). Clarifying this distinction may lead to more accurate diagnostic practices and more effective intervention strategies.

Beyond phenomenological and neurocognitive overlap, converging evidence from psychiatric genetics indicates a substantial shared genetic liability between ASD and ADHD. Large-scale genome-wide association studies (GWAS) have demonstrated significant genetic correlations between the two conditions, suggesting partially shared polygenic architectures. Recent cross-disorder genomic analyses (13; doi: 10.1038/s41588-022-01171-3) show that common variants associated with neurodevelopmental and psychiatric traits often confer risk across traditional diagnostic boundaries, including ASD and ADHD.

Moreover, emerging large-scale genomic studies (doi: 10.1038/s41586-025-0942-6) highlight how pleiotropic genetic effects influence multiple neurodevelopmental phenotypes, supporting a dimensional view of neurodevelopmental liability. Importantly, while a significant proportion of genetic risk is shared, disorder-specific genetic influences also remain, which may contribute to the heterogeneity of clinical presentations. This genetic perspective aligns with dimensional and transdiagnostic models of psychopathology and provides a biological basis for investigating ASD+ADHD as a potentially distinct or partially overlapping phenotype rather than a mere diagnostic coincidence.

From a cognitive standpoint, both ASD and ADHD are associated with executive functioning difficulties, although their profiles differ. Meta-analytic studies have shown broad Executive Functioning (EF) impairments in ASD, including difficulties in cognitive flexibility, inhibition, and planning (14–21). ADHD, instead, is typically characterized by prominent deficits in inhibitory control, sustained attention, and attentional regulation (22, 23).

Behaviorally, ADHD is more often associated with externalizing symptoms such as aggression and rule-breaking, whereas ASD tends to involve internalizing traits like social withdrawal and affective flattening (24–26).

The Child Behavior Checklist (CBCL; 27) is widely used to assess these behavioral-emotional patterns and has shown strong utility in distinguishing clinical subtypes in neurodevelopmental populations (28–33). Recent models, such as the predictive coding theory and hypo-prior hypothesis, offer neurocognitive frameworks that help explain atypical sensory and social processing in ASD, and may also be extended to comorbid presentations (32, 34–37).

Despite high comorbidity rates, ASD+ADHD presentations remain under-investigated. A key reason is historical: until DSM-5, concurrent diagnoses of ASD and ADHD were not permitted, limiting scientific inquiry. Furthermore, research has often focused on single-disorder samples, leaving comorbid profiles relatively unexplored. This has resulted in a limited understanding of whether ASD+ADHD reflects a discrete phenotype or merely the additive expression of two neurodevelopmental conditions.

aThe current study aims to systematically compare the cognitive and emotional-behavioral profiles of children with ASD, ADHD, and comorbid ASD+ADHD using two standardized tools: the Wechsler Intelligence Scale for Children – Fourth Edition (WISC-IV; 38) and the CBCL 6–18. Previous studies suggest that stronger cognitive abilities—particularly verbal comprehension and working memory—may act as protective factors for behavioral and emotional regulation in ASD and ADHD (39, 40). This provides a rationale for examining whether such associations are preserved or disrupted in ASD+ADHD comorbidity.

We hypothesize that the ASD+ADHD group will exhibit (1) significantly lower scores in working memory, processing speed, and full-scale IQ, and (2) a broader behavioral-emotional dysregulation profile, including elevated internalizing and externalizing symptoms. Furthermore, we expect that cognitive abilities will show protective associations with behavioral outcomes in the ASD and ADHD groups, but not in the ASD+ADHD group, reflecting disrupted cognitive-emotional integration in the comorbid phenotype.

## 2 Materials and methods

### 2.1 Participants

The sample consisted of 207 children and adolescents (35 girls and 172 boys; ages 6–16) referred for neurodevelopmental evaluation. Participants were divided into three clinical groups: Autism Spectrum Disorder (ASD; n = 21; 4 girls and 17 boys; mean age: 11.53 ± 3.39 years), Attention- Deficit/Hyperactivity Disorder (ADHD; n = 103; 15 girls and 88 boys; mean age: 11.02 ± 2.57 years), and ASD+ADHD comorbidity (n = 83; 16 girls and 67 boys; mean age: 10.22 ± 2.59 years).

Diagnoses were made according to DSM-5 criteria by multidisciplinary teams at the IRCCS Stella Maris Foundation, Calambrone (Pisa). Diagnostic procedures included structured clinical interviews with caregivers, direct behavioral observation of the child, and review of developmental and medical history. When available, standardized instruments such as Autism Diagnostic Observation Schedule-2 (ADOS-2; 41), Autism Diagnostic Interview-Revised (ADI-R; 42), or Conners scales (43) were integrated to support diagnostic decisions. Clinicians involved were specialists in neurodevelopmental disorders with extensive experience in ASD and ADHD assessment. All participants were Italian speakers with normal or corrected-to-normal vision and hearing. Inclusion criteria were: (a) age between 6 and 16 years; (b) complete Wechsler Intelligence Scale for Children-IV (WISC-IV; 38) and Child Behavior Check List (CBCL; 27) data; and (c) confirmed diagnosis of ASD, ADHD, or ASD+ADHD based on multidisciplinary evaluation. Because only cases with complete WISC-IV and CBCL data were included, no missing data procedures or imputations were necessary.

Exclusion criteria included neurological conditions, genetic syndromes, or incomplete diagnostic documentation. Intellectual disability (Full Scale Intelligence Quotient FSIQ < 70) was not an exclusion criterion unless accompanied by severe adaptive impairments.

### 2.2 Instruments

• WISC-IV (Wechsler Intelligence Scale for Children – Fourth Edition; 38; Italian version by 44): a standardized tool used to assess general intellectual functioning in children. It yields a Full-Scale IQ (FSIQ) as an overall measure of cognitive ability, along with four primary index scores: Verbal Comprehension (VCI), Perceptual Reasoning (PRI), Working Memory (WMI), and Processing Speed (PSI). These indices provide a detailed profile of cognitive strengths and weaknesses across verbal, non-verbal, memory-related, and speed-based domains.
• CBCL 6-18 (Child Behavior Checklist; 27; Italian adaptation by 45): a standardized parent-report questionnaire designed to assess a wide range of behavioral and emotional difficulties in children and adolescents. across three main domains – Syndrome scales (Anxious/Depressed, Withdrawn/Depressed, Somatic Complaints, Social Problems, Thought Problems, Attention Problems, Rule-Breaking Behavior, Aggressive Behavior), Competence scales (Activities, Social, School), and DSM- oriented scales (Affective Problems, Anxiety Problems, Somatic Problems, Attention Deficit/Hyperactivity Problems, Oppositional Defiant Problems, Conduct Problems). Raw scores obtained from the CBCL were converted into standardized T-scores, allowing for comparison against normative data.

### 2.3 Procedure and statistical approach

Data were retrospectively collected from clinical records and structured assessments conducted between 2017 and 2024. The reporting of this study was aligned, where applicable, with the RECORD (REporting of studies Conducted using Observational Routinely-collected health Data) guidelines to enhance transparency and methodological rigor. The WISC-IV was administered by licensed psychologists trained in standardized cognitive assessment. Caregivers completed the CBCL. Statistical analyses were performed using SPSS version 29.0. One-way ANoVAs were conducted to examine potential differences between diagnostic groups (ASD, ADHD, and ASD+ADHD) in cognitive measures and emotional-behavioral profiles. When significant group effects were found, Bonferroni-corrected *post hoc* comparisons were applied to identify specific group differences. Additionally, Pearson correlation analyses were carried out separately within each group to explore significant associations between cognitive and emotional-behavioral variables, providing insight into group-specific relationships among these measures. Prior to analysis, distributional assumptions were inspected, and variables showed approximately normal distributions. Given the sample sizes and the robustness of Pearson’s coefficient to moderate deviations from normality, Pearson correlations were considered appropriate. Covariates such as age and sex were not included in statistical models to preserve power in the smallest group (ASD) and because of the retrospective design.

## 3 Results

Preliminary analyses on demographic variables were conducted using a one-way ANOVA and chi-square tests. The ANOVA indicated a marginal age difference among groups, F(2,204) = 3.05, p = .051, with the ASD+ADHD group being slightly younger on average. A chi-square test showed that sex distribution did not significantly differ among groups, χ²(2) = 0.80, p = .669.

### 3.1 Cognitive profile (WISC-IV)

One-way ANOVAs revealed no significant group differences for the Verbal Comprehension Index, F(2, 204) = 1.98, p = .140, or for the Perceptual Reasoning Index, F(2, 204) = 2.57, p = .079.

A significant group effect emerged for the Working Memory Index, F(2, 204) = 3.61, p = .029. *Post hoc* comparisons indicated that the ASD group scored significantly higher than the ASD+ADHD group (p = .024). Similarly, the Processing Speed Index showed significant group differences, F(2, 204) = 4.74, p = .010, with the ASD group again outperforming the ASD+ADHD group (p = .015).

For Full Scale IQ, a significant main effect of group was observed, F(2, 204) = 6.49, p = .002. Bonferroni-adjusted comparisons revealed that the ASD group had significantly higher FSIQ scores than both the ADHD (p = .004) and ASD+ADHD (p = .001) groups.

The ASD+ADHD group showed significantly lower scores than the ASD group but did not differ significantly from the ADHD group, indicating a cognitive profile more similar to ADHD than to ASD.

The effect sizes ranged from small to medium (η² = .034–.062), with the largest effect observed for Full-Scale IQ, suggesting a meaningful difference between groups in global cognitive functioning (see **Table 1**).

**Table 1:** WISC-IV scores by diagnostic group.

	ASD (n=21)	ADHD (n=103)	ASD+ADHD (n=83)	*F* (2, 204)	*p*	*H^2^*	Group significance
WISC-IV	*M (SD)*	*M (SD)*	*M (SD)*				
Verbal Comprehension	108.00 (26.26)	98.24 (20.06)	96.87 (25.65)	1.983	.140	.019	N.S.
Processing Speed	104.00 (28.18)	93.16 (20.40)	91.40 (24.33)	2.571	.079	.025	N.S.
Working Memory	97.67 (25.08)	84.72 (22.23)	82.07 (25.34)	3.605	.029	.034	ASD>ASD+ADHD
Processing Speed	88.81 (24.20)	81.31 (17.89)	75.36 (19.89)	4.743	.010	.044	ASD>ASD+ADHD
Full Scale IQ	107.79 (22.93)	88.28 (22.18)	86.24 (25.86)	6.494	.002	.062	ASD>ADHD, ASD+ADHD

### 3.2 Behavioral and emotional profile (CBCL 6-18)

Concerning the CBCL 6–18 Syndrome Scales, a significant group effect emerged for Withdrawn/Depressed, F(2, 204) = 6.41, p = .002. *Post hoc* analyses indicated that participants with ASD showed significantly higher scores compared to those with ADHD (p = .003), suggesting greater difficulties related to social withdrawal and low mood in the ASD group. Significant differences were also found for Rule-Breaking Behavior, F(2, 204) = 8.51, p <.001, and Aggressive Behavior, F(2, 204) = 8.92, p <.001, with the ADHD group reporting significantly higher scores than both the ASD (p = .003 and p = .004, respectively) and ASD+ADHD groups (p = .005 and p = .002, respectively). With regard to the Broad-Band Scales, a significant effect was observed for Externalizing Problems, F(2, 204) = 11.15, p <.001. The ADHD group displayed higher levels of externalizing symptoms compared to both the ASD (p = .001) and ASD+ADHD groups (p = .001). No significant differences were found for Internalizing or Total Problems.

As for the DSM-Oriented Scales, group differences emerged for ADHD Problems, F(2, 204) = 9.26, p <.001, with higher scores in both the ADHD and ASD+ADHD groups relative to ASD (p <.001 and p = .001 respectively) participants. Similarly, Oppositional Defiant Problems, F(2, 204) = 6.68, p = .002, and Conduct Problems, F(2, 204) = 11.49, p <.001, were more elevated in the ADHD group compared to both ASD (p = .009 and p = .004, respectively) and ASD+ADHD (p = .015 and p <.001, respectively).

Finally, significant group effects were found for two Supplementary Scales. Sluggish Cognitive Tempo, F(2, 204) = 7.35, p = .001, was significantly higher in both the ASD and ASD+ADHD groups compared to ADHD (p = .015 and p = .004 respectively) group. Similarly, Obsessive–Compulsive Problems, F(2, 204) = 6.02, p = .003, were more prominent in the ASD and ASD+ADHD groups compared to ADHD (p = .007 and p = .050 respectively).

In terms of effect size, the most substantial differences were observed for Externalizing Problems (η²= .099), Conduct Problems (η² = .101), and Aggressive Behavior (η² = .080), indicating that these behavioral domains carry not only statistical but also clinical weight in differentiating the groups. For the remaining scales and domains—including competence scales, internalizing symptoms, and several syndrome and DSM-oriented subscales—no statistically significant group differences were found. Detailed descriptive and inferential statistics for all CBCL domains are reported in **Table 2**. No significant group differences emerged on the CBCL Total Problems scale, indicating that overall behavioral symptom severity did not differ significantly across groups (see **Table 1**).

**Table 2:** CBCL 6–18 scores by diagnostic group.

CBCL 6–18 Scales	ASD	ADHD	ASD+ADHD	F	*P*	η²	Group
	(n=21)	(N = 103)	(N = 83)				Significance
	M (SD)	M (SD)	M (SD)				
Competence scales	36,60 (10,32)	33.41 (9.09)	32.59 (10.36)	0.938	.395	.017	N.S.
Activities							
Social Functioning	31,60 (9,48)	36.97 (9.13)	33.77 (7.65)	3.005	.054	.052	N.S.
School	39,93 (8,54)	36.33 (7.61)	35.58 (7.17)	1.770	.175	.034	N.S.
Total Competence	30,20 (10,79)	29.86 (8.54)	26.91 (7.34)	1.418	.247	.027	N.S.
Syndrome scales							
Anxious/Depressed	67.95 (10,07)	65.37 (10.02)	66.69 (10.19)	0.760	.469	.007	N.S.
Withdrawn/Depressed	73.10 (14,20)	64.42 (9.32)	67.65 (11.28)	6.412	.002	.059	ASD>ADHD
Somatic Complaints	59.38 (10,09)	59.59 (7.87)	60.05 (7.93)	0.097	.908	.001	N.S.
Social Problems	67.86 (8,97)	66.71 (9.34)	67.14 (10.09)	0.140	.869	.001	N.S.
Thought Problems	69.71 (11,30)	65.05 (10.31)	67.61 (10.81)	2.378	.095	.023	N.S.
Attention Problems	66.33 (12,10)	69.21 (10.28)	71.43 (11.15)	2.171	.117	.021	N.S.
Rule-Breaking Behaviour	57.24 (6,11)	63.85 (8.52)	59.99 (8.18)	8.509	<.001	.077	ADHD> ASD, ASD+ADHD
Aggressive Behaviour	61.95 (10,60)	70.45 (11.37)	64.75 (10.62)	8.924	<.001	.080	ADHD> ASD, ASD+ADHD
Broad-band scales							
Internalizing Problems	67.52 (11.29)	64.75 (8.83)	66.59 (8.83)	1.375	.255	.013	N.S.
Externalizing Problems	59.57 (8.95)	67.72 (8.54)	62.55 (9.91)	11.146	<.001	.099	ADHD> ASD, ASD+ADHD
Total Problems	66.76 (8.72)	69.06 (8.12)	67.90 (8.63)	0.860	.425	.008	N.S.
DSM-oriented scales							
Affective Problems	68.86 (9.94)	67.42 (10.04)	68.93 (9.73)	0.590	.555	.006	N.S.
Anxiety Problems	67.14 (8.43)	64.83 (7.47)	66.70 (7.23)	1.812	.166	.017	N.S.
Somatic Problems	55.33 (9.15)	57.63 (7.85)	56.51 (8.10)	0.909	.405	.009	N.S.
ADHD	60.05 (8.65)	68.60 (8.00)	67.48 (8.62)	9.256	<.001	.083	ASD< ADHD, ASD+ADHD
Oppositional Defiant Problems	60.00 (7.21)	66.25 (8.82)	62.60 (8.92)	6.680	.002	.061	ADHD>ASD, ASD+ADHD
Conduct Problems	59.00 (7,75)	65.97 (9.19)	60.40 (8.70)	11.492	<.001	.101	ADHD> ASD, ASD+ADHD
Supplementary scales							
Sluggish Cognitive Tempo	66.29 (8.70)	60.40 (8.50)	64.54 (8.78)	7.347	.001	.010	ADHD<ASD, ASD+ADHD
Obsessive-Compulsive	71.24 (12.57)	63.06 (10.68)	67.02 (11.37)	6.017	.003	.067	ADHD<ASD, ASD+ADHD
Post-Traumatic Stress	71.76 (11.63)	68.95 (9.19)	70.13 (10.13)	0.839	.433	.008	N.S.

P= <.005.

### 3.3 Correlational analyses

**Table 3** presents the Pearson correlation coefficients between cognitive profile measures (WISC-IV indexes) and behavioral and emotional profile scores (CBCL 6–18 subscales) in the ADHD group. Significant negative correlations were found between several WISC-IV indexes and CBCL domains reflecting behavioral and emotional difficulties. In particular, higher VCI scores were associated with lower levels of Anxious/Depressed (r = –.207), Withdrawn/Depressed (r = –.285), Social Problems (r = –.427), Attention Problems (r = –.331), Internalizing Problems (r = –.206), Total Problems (r = –.194), Affective Problems (r = –.258), Anxiety Problems (r = –.243), ADHD Problems (r= –.201), Sluggish Cognitive Tempo (r = –.249), and Post-Traumatic Stress (r = –.210).

**Table 3:** Pearson correlations between cognitive profile measures (WISC-IV indexes) and behavioral and emotional profile scores (CBCL 6–18 subscales) in the ADHD group (N = 103).

Measure	VCI	PRI	WMI	PSI	FSIQ
Competence scales					
Activities	.143	.071	.069	.134	.113
Social Functioning	.098	.053	.035	.026	.071
School	.206	.236	.362**	.238	.297*
Total Competence	.262	.205	.170	.204	.253
Syndrome scales					
Anxious/Depressed	-.207*	-.050	-.176	-.080	-.115
Withdrawn/Depressed	-.285**	-.149	-.275**	-.008	-.218*
Somatic Complaints	-.074	-.006	-.111	-.079	-.042
Social Problems	-.427**	-.282**	-.380**	-.383**	-.434**
Thought Problems	-.061	.043	-.025	-.178	-.025
Attention Problems	-.331**	-.224*	-.261**	-.289**	-.311**
Rule-Breaking Behavior	-.103	-.017	.057	-.014	-.034
Aggressive Behavior	-.103	.018	.003	-.078	-.042
Broad-band scales					
Internalizing Problems	-.206*	-.035	-.177	-.064	-.119
Externalizing Problems	-.104	.013	.028	-.067	-.041
Total Problems	-.194*	-.065	-.114	-.142	-.140
DSM-oriented scales					
Affective Problems	-.258**	-.117	-.220*	-.174	-.198*
Anxiety Problems	-.243*	-.097	-.242*	-.170	-.190
Somatic Problems	.032	.061	-.018	.010	.060
ADHD Problems	-.201*	-.097	-.173	-.160	-.175
Oppositional Defiant Problems	-.034	.051	.027	-.071	.005
Conduct Problems	-.110	-.027	.033	-.016	-.048
Supplementary scales					
Sluggish Cognitive Tempo	-.249*	-.141	-.220*	-.184	-.209*
Obsessive–Compulsive	-.174	-.069	-.080	-.167	-.119
Post-Traumatic Stress	-.210*	-.053	-.150	-.140	-.121

*p <.05. **p <.01.

The PRI was negatively correlated with Social Problems (r = –.282) and Attention Problems (r = –.224), though no other significant associations were observed.

Regarding the WMI, higher scores were associated with lower levels of School Problems (r = .362), Withdrawn/Depressed (r = –.275), Social Problems (r = –.380), Attention Problems (r = –.261), Affective Problems (r = –.220), Anxiety Problems (r = –.242) and Sluggish Cognitive Tempo (r = –.220).

The PSI was negatively correlated with Social Problems (r = –.383) and Attention Problems (r = –.289), while the FSIQ showed significant negative correlations with Social Problems (r = –.434), Attention Problems (r = –.311), Withdrawn/Depressed (r = –.218), Affective Problems (r = –.198), and Sluggish Cognitive Tempo (r = –.209). A positive association emerged between FSIQ and School competence (r = .297).

Overall, stronger cognitive abilities—particularly in verbal comprehension, working memory, and global functioning—were associated with lower behavioral and emotional problems, especially in the domains of attention, social functioning, and mood-related symptoms.

As for the ASD group, the results of the correlation analyses are summarized in **Table 4**. Significant positive correlations were found between cognitive measures and school functioning. In particular, higher scores on the VCI were associated with better performance in the School domain (r = .661). Similarly, PRI, WMI, and FSIQ scores were positively related to School performance (r >.61), suggesting that overall cognitive functioning is closely linked to academic outcomes in children with ASD. Conversely, significant negative correlations emerged between cognitive indexes and various behavioral and emotional difficulties. VCI, PRI, WMI, and FSIQ showed consistent negative associations with Social Problems (r > –.51). Attention Problems were negatively associated with all cognitive indexes (r > –.49). In addition, Total Problems were negatively correlated with VCI, PRI, and WMI scores (r = –.628, –.647, –.700, respectively). These findings indicate that lower verbal, perceptual, and working memory skills are strongly associated with greater behavioral symptoms in children with ASD.

**Table 4:** Pearson correlations between cognitive profile measures (WISC-IV indexes) and behavioral and emotional profile scores (CBCL 6–18 subscales) in the ASD group (N = 21).

Measure	VCI	PRI	WMI	PSI	FSIQ
Competence scales					
Activities	-.020	-.235	-.108	-.211	.159
Social Functioning	.490	.342	.441	.243	.362
School	.661**	.606*	.681**	.492	.810**
Total Competence	.361	.176	.301	.087	.399
Syndrome scales					
Anxious/Depressed	-.230	-.405	-.278	-.119	-.165
Withdrawn/Depressed	-.259	-.255	-.439*	-.075	.011
Somatic Complaints	-.517*	-.624**	-.628**	-.401	-.158
Social Problems	-.534*	-.673**	-.735**	-.333	-.510*
Thought Problems	-.485*	-.522*	-.458*	-.511*	-.305
Attention Problems	-.686**	-.720**	-.749**	-.493*	-.552*
Rule-Breaking Behavior	-.571**	-.301	-.485*	-.427	-.298
Aggressive Behavior	-.608**	-,453*	-.443*	-.380	-.257
Broad-band scales					
Internalizing Problems	-.386	-.477*	-.556**	-.140	-.222
Externalizing Problems	-.618**	-.424	-.476*	-.352	-.330
Total Problems	-.628**	-.647**	-.700**	-.392	-.442
DSM-oriented scales					
Affective Problems	-.352	-.388	-.462*	-.254	-.186
Anxiety Problems	-.253	-.393	-.303	-.162	-.219
Somatic Problems	-.495*	-.550**	-.605**	-.400	-.203
ADHD Problems	-.535*	-.614**	-.605**	-.374	-.370
Oppositional Defiant Problems	-.678**	-.549**	-.527*	-.478*	-.412
Conduct Problems	-.532*	-.323	-.349	-.370	-.226
Supplementary scales					
Sluggish Cognitive Tempo	-.221	-.362	-.321	-.176	-.013
Obsessive–Compulsive	-.398	-.570**	-.435*	-.415	-.406
Post-Traumatic Stress	-.528*	-.589**	-.594**	-.236	-.349

*p <.05. **p <.01.

Furthermore, significant correlations were observed between WMI and internalizing domains, including Somatic Complaints (r = –.628), Internalizing Problems (r = –.556), and Affective Problems (r = –.462). Negative associations were also found between PSI and several behavioral outcomes, such as Attention Problems (r = –.493) and Oppositional Defiant Problems (r = –.478).

Overall, the data suggest that higher cognitive functioning in children with ASD is associated with better academic and social outcomes, as well as fewer behavioral and emotional difficulties— particularly those involving attention, somatic symptoms, and social interaction.

Considering the ASD+ADHD group, the results of the correlation analyses are reported in **Table 5**. The results show fewer significant associations compared to the other clinical groups. However, a moderate positive correlation was found between the PRI and School performance (r = .466), as well as between the WMI and School (r = .434), indicating that stronger perceptual and working memory abilities are linked to better academic functioning in children with comorbid ASD and ADHD. Additionally, a small but significant correlation emerged between FSIQ and Total Competence (r =.378), suggesting that higher overall cognitive functioning may support general adaptive abilities in this group.

**Table 5:** Pearson correlations between cognitive profile measures (WISC-IV indexes) and behavioral and emotional profile scores (CBCL 6–18 subscales) in the ASD+ADHD group (N = 83).

Measure	VCI	PRI	WMI	PSI	FSIQ
Competence scales					
Activities	.027	-.028	.057	.010	.228
Social Functioning	.047	.092	.088	.089	.256
School	.266	.466**	.434*	.114	.248
Total Competence	.081	.151	.230	.154	.378*
Syndrome scales					
Anxious/Depressed	.014	-.072	.090	.111	.013
Withdrawn/Depressed	.139	.114	.101	.127	.096
Somatic Complaints	.160	.157	.169	.191	.204
Social Problems	-.010	.035	.025	.033	.058
Thought Problems	.071	-.005	.194	.078	.105
Attention Problems	-.083	-.135	-.038	.031	-.034
Rule-Breaking Behavior	.054	-.059	.090	.029	.032
Aggressive Behavior	.197	.043	.206	.137	.155
Broad-band scales					
Internalizing Problems	.136	.032	.171	.149	.120
Externalizing Problems	.110	-.017	.149	.063	.079
Total Problems	.090	-.022	.166	.104	.107
DSM-oriented scales					
Affective Problems	.128	.052	.157	.158	.101
Anxiety Problems	-.055	-.161	-.072	-.113	-.070
Somatic Problems	.137	.046	.105	.188	.145
ADHD Problems	-.019	-.080	.080	.028	.027
Oppositional Defiant Problems	.231*	.081	.208	.160	.202
Conduct Problems	.164	-.018	.166	.137	.104
Supplementary scales					
Sluggish Cognitive Tempo	.166	.099	.138	.146	.130
Obsessive–Compulsive	.034	-.090	.136	.089	.010
Post-Traumatic Stress	.113	-.043	.099	.085	.070

*p <.05. **p <.01.

Interestingly, VCI showed a weak but significant positive correlation with Oppositional Defiant Problems (r = .231), which contrasts with findings in other groups where higher cognitive scores were generally associated with fewer behavioral symptoms. This unexpected result may reflect unique behavioral dynamics in the comorbid group and warrants further investigation.

## 4 Discussion

The pattern observed in the ASD+ADHD group reflects a combination of ASD- and ADHD-related characteristics. Although certain group differences emerged, the overlap in cognitive performance with the ADHD group and the absence of differences in overall behavioral severity suggest that conclusions about a distinct phenotype should be drawn cautiously. Consistent with our hypotheses, the ASD+ADHD group showed lower cognitive performance than the ASD group; however, their scores did not differ significantly from the ADHD group. It is important to note that age differed slightly across groups; however, this difference was small in magnitude (approximately one year) and all WISC-IV and CBCL scores were age-standardized, reducing the likelihood that age significantly influenced the observed group differences. These findings align with prior work showing that the co- occurrence of ASD and ADHD results in amplified executive dysfunction, with medium to large effect sizes supporting their clinical relevance (14, 46–49).

Behaviorally, the ADHD group presented with prominent externalizing behaviors, such as aggression and rule-breaking, in line with disinhibition models of ADHD (22, 50, 51). The ASD group, conversely, exhibited higher levels of withdrawn/depressed traits, reflecting internalizing affective symptoms (26, 52). The comorbid ASD+ADHD group demonstrated the broadest spectrum of dysregulation, encompassing both internalizing and externalizing domains, as well as elevated scores on supplementary CBCL scales such as Sluggish Cognitive Tempo and Obsessive–Compulsive symptoms (53, 54).

One of the most striking findings is the relative absence of protective correlations between cognitive functioning and behavioral outcomes in the ASD+ADHD group, in contrast to the other two groups. While verbal and working memory skills in ASD and ADHD were significantly associated with better school functioning and fewer emotional-behavioral symptoms, these associations were diminished or reversed in the comorbid group. This dissociation may reflect a breakdown in the cognitive- emotional integration mechanisms that typically support adaptive functioning in neurodevelopmental populations (18, 40, 55).

An additional point worth considering concerns the interpretation of attentional difficulties in children with ASD, particularly those with higher cognitive abilities. Recent literature highlights the ongoing challenges in differentiating ADHD-related inattention from attentional patterns associated with ASD-related cognitive styles (56). In individuals with ASD, apparent inattention may sometimes reflect differences in information processing, selective interest, or reduced engagement with non-preferred stimuli rather than primary executive dysfunction. This distinction is clinically relevant, as it underscores the importance of integrating multiple sources of information—including clinical observation, developmental history, and neuropsychological assessment—when evaluating attentional symptoms. Such a multimethod approach may help avoid over- or under-identification of ADHD in cognitively able autistic individuals and may partly explain variability in school-related outcomes.

These results may be cautiously considered within the framework of predictive processing theories, which have been proposed to explain atypical sensory and perceptual inference in ASD (34, 36, 57). ADHD, in contrast, is characterized primarily by attentional dysregulation rather than alterations in predictive coding (58). In comorbid ASD+ADHD, the interaction between atypical perceptual inference and attentional control difficulties may contribute to challenges in behavioral and emotional regulation; however, this interpretation remains speculative and requires direct neurocognitive investigation.

This interpretation is consistent with recent findings on altered cortical connectivity and reduced error prediction in comorbid samples (59).

From a clinical standpoint, these findings underscore the necessity of recognizing ASD+ADHD comorbidity as a distinct phenotype that requires specific assessment and intervention approaches. Comprehensive evaluations should integrate cognitive testing (e.g., WISC-IV) with dimensional behavioral measures (e.g., CBCL) to capture the full range of functioning. Intervention protocols should be multimodal, combining executive function training with behavioral regulation strategies, emotional resilience building, and family psychoeducation. Importantly, approaches that capitalize on cognitive strengths—often effective in ASD—may be less generalizable to comorbid cases.

**Figure 1:**
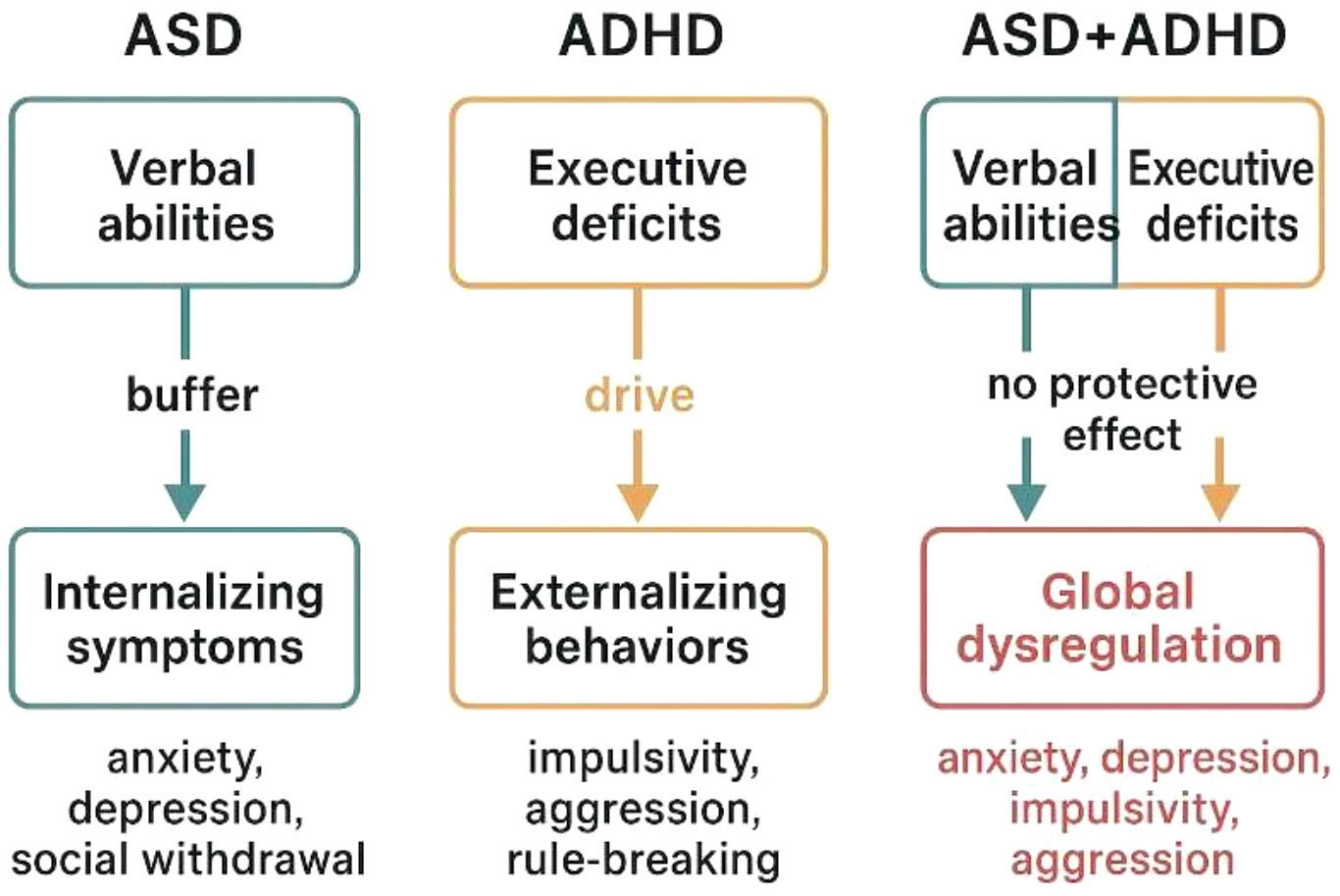
Heuristic model of cognitive-behavioral profiles in ASD, ADHD, and ASD+ADHD comorbidity.

We propose a heuristic model (**Figure 1**) to visually represent the distinct cognitive-behavioral integration patterns across groups. In ASD, higher verbal skills buffer internalizing symptoms; in ADHD, executive deficits drive externalizing behavior. In ASD+ADHD, executive and regulatory systems appear jointly compromised, disrupting this buffering mechanism and contributing to more global dysregulation. This heuristic model can also be viewed in relation to other contemporary frameworks proposed to understand neurodevelopmental comorbidity. Recent models emphasize transdiagnostic dimensions, shared neurocognitive liabilities, and the dynamic interaction between cognitive control, emotional regulation, and environmental demands (60, 61). Within this perspective, our model aligns with dimensional approaches by highlighting both shared and disorder-specific mechanisms, while also proposing that the co-occurrence of ASD and ADHD may involve non-additive interactions that alter the typical protective role of cognitive strengths. Thus, rather than competing with existing models, the present heuristic framework may be seen as a clinically grounded complement that integrates cognitive and behavioral data at the phenotypic level.

These findings suggest that dimensional and phenotype-informed approaches may offer useful insights for understanding comorbidity, although further research with more comprehensive designs is needed before drawing firm conclusions. Future longitudinal studies are needed to explore developmental trajectories and treatment responsiveness in comorbid presentations. Integration of neurobiological, cognitive, and behavioral data will be essential to advance precision psychiatry for neurodivergent youth (36, 39, 57, 59, 62).

### 4.1 Limitations

Several limitations should be considered when interpreting the present findings. First, the sample size of the ASD-only group was relatively small compared to the ADHD and ASD+ADHD groups, which may have affected the statistical power to detect more subtle differences. However, this distribution mirrors real-world referral patterns in clinical neurodevelopmental settings. Second, the study used a cross-sectional and retrospective design based on clinical data, which may be susceptible to selection and information biases. Third, the reliance on parent-report behavioral assessments (CBCL) limits the ecological validity of the findings; future research should incorporate multi-informant approaches, including teacher reports and direct behavioral observations. Finally, the absence of biological or neuroimaging data prevents definitive conclusions about the neural mechanisms underlying the observed profiles. Future studies integrating behavioral, cognitive, and neurobiological data will be crucial to refine diagnostic classification and personalize interventions. Additional limitations include the absence of socioeconomic and ethnic data, lack of standardized diagnostic instruments for all participants, and the decision not to include covariates such as age, sex, or medication status in statistical models. Moreover, although sex distribution did not significantly differ among groups, we did not specifically investigate sex-related effects. This is a relevant limitation, as increasing evidence indicates that sex differences can significantly influence the clinical presentation, symptom expression, and developmental trajectories of both ASD and ADHD, as well as their comorbid manifestations (63). Future studies should explicitly examine sex-specific patterns to better capture the heterogeneity of neurodevelopmental conditions. These factors limit generalizability and may have influenced observed group differences.

## 5 Conclusion

This study contributes to the growing body of literature emphasizing the need to conceptualize ASD+ADHD comorbidity as a distinct clinical and cognitive phenotype, rather than a simple additive overlap of two separate disorders. Children with comorbid presentations showed lower cognitive performance than the ASD group but did not differ significantly from the ADHD group, and displayed intermediate behavioural patterns across CBCL domains.

While these findings emerge from an Italian clinical sample, the observed cognitive and emotional profiles are likely relevant to broader neurodevelopmental populations, given the use of standardized, cross-culturally validated assessment tools.

Moreover, the comorbid group showed greater severity in supplementary behavioral dimensions, such as Sluggish Cognitive Tempo and Obsessive–Compulsive symptoms, which aligned more closely with the ASD phenotype. These findings suggest that comorbidity may involve both the accumulation and amplification of vulnerabilities associated with each individual disorder.

The data also confirm that verbal cognitive abilities may act as a protective factor in children with ASD, facilitating better academic adaptation and reduced behavioral difficulties, particularly in the domains of attention, emotional regulation, and social interaction. However, this protective association was markedly attenuated—or altogether absent—in the ASD+ADHD group, where cognitive performance showed limited and inconsistent correlations with emotional-behavioral outcomes. This suggests a disintegration of the regulatory role of cognitive strengths in comorbid presentations, potentially due to the simultaneous disruption of attentional control, inhibition, and predictive processing systems.

From a clinical perspective, these findings highlight the importance of early, comprehensive, and multidimensional assessment strategies—particularly for children with suspected dual diagnoses. Tailored interventions should address executive dysfunction, emotional and behavioral dysregulation, and adaptive skill development. Furthermore, capitalizing on individual cognitive strengths, where present, may support compensatory mechanisms in ASD, though such approaches may require substantial adaptation when addressing comorbid cases.

The reporting of effect sizes alongside p-values supports a more nuanced interpretation of the findings and aligns with best practices in clinical research reporting.

Future research should pursue longitudinal designs to track developmental trajectories and examine how cognitive-behavioral profiles shift over time across diagnostic groups. Neurobiological investigations—including structural and functional neuroimaging, electrophysiology, and genetic studies—may elucidate the shared and unique mechanisms underlying ASD, ADHD, and their intersection. Additionally, incorporating environmental and contextual factors (e.g., family dynamics, school supports, socio-economic status) will be essential in identifying modifiable protective factors and enhancing real-world functional outcomes.

Ultimately, embracing the complexity and heterogeneity inherent to neurodevelopmental conditions like ASD and ADHD—and their frequent comorbidity—will enhance our ability to provide individualized, evidence-based care. A precision clinical approach that considers both dimensional and categorical models of functioning is critical to addressing the diverse needs of neurodivergent individuals and their families.

## Data Availability

The raw data supporting the conclusions of this article will be made available by the authors, without undue reservation.
